# Synthesis and Characterization of GdVO_4_:Nd Near-Infrared Phosphors for Optical Time-Gated In Vivo Imaging

**DOI:** 10.3390/ma13163564

**Published:** 2020-08-12

**Authors:** Ben Nimmegeers, Ewoud Cosaert, Tecla Carbonati, Daniela Meroni, Dirk Poelman

**Affiliations:** 1 LumiLab, Department of Solid State Sciences, Ghent University, 9000 Ghent, Belgium; ben.nimmegeers@ugent.be (B.N.); ewoud.cosaert@ugent.be (E.C.); 2 Nanomaterials and Interfaces Group, Dipartimento di Chimica, Universita degli Studi di Milano, 20122 Milano, Italy; tecla.carbonati@studenti.unimi.it (T.C.); daniela.meroni@unimi.it (D.M.)

**Keywords:** near-infrared luminescence, multimodal imaging, bio-imaging, time-gated detection, neodymium, Nd^3+^

## Abstract

Many medical imaging techniques use some form of ionizing radiation. This radiation is not only potentially harmful for the patient, but also for the medical personnel. An alternative imaging technique uses near-infrared (NIR) emitting luminescent particles as tracers. If the luminescent probes are excited inside the body, autofluorescence from the biological tissues is also induced. This problem can be circumvented by using time-gated imaging. Hereby, the light collection only starts when the fluorescence of the tissue has decayed. This requires particles showing both excitation and emission in the near-infrared and a long decay time so that they can be used in time-gated imaging. In this work, Nd-doped GdVO${}_{4}$ NIR emitting particles were prepared using solid state reaction. Particles could be efficiently excited at 808 nm, right in the first transparency window for biological tissues, emitted in the second transparency window at around 1064 nm, and showed a decay time of the order of 70 μs, sufficiently long for time-gating. By using a Gd-containing host, these particles could be ideally suited for multimodal optical/magnetic imaging after size reduction and surface functionalization.

## 1. Introduction

There are many existing medical techniques to visualize the human body. Most of them use high energy photons. Those photons can penetrate the body so that an image can be taken. X-ray radiography and CT (computed tomography) are the techniques that are used to form a 2D or 3D image of the body for anatomic information of the patient. PET (positron emission tomography) and SPECT (single photon emission computed tomography) provide functional information by adding a radioactive tracer to a functional molecule, called the pharmakon. Depending on the type and functionality of the pharmakon, this will target a specific part of the body, like a tumor. The radiation of this radioactive molecule is harmful for the patient and everyone who comes close to the patient [1], especially when imaging is performed during surgery. Beside those invasive tools, there are imaging techniques that are harmless for the patient and surroundings, like MRI (magnetic resonance imaging) and ultrasound. MRI brings the proton densities into view; however, due to the high magnetic field that is required, this technique is expensive, and there is limited access to MRI instruments. Ultrasound is only suitable for imaging of organs at the surface of the body, and the image quality is much lower than that of other techniques.

Just like high energy radiation, near-infrared (NIR) radiation of a suitable wavelength has a low absorption in the body, but its potential for medical imaging has not been fully explored so far. The interest in this NIR region is growing, with the aim to find other imaging methods that are harmless for the patient and medical staff. To use NIR imaging, luminescent particles are needed that emit in the NIR. Organic NIR emitting molecules such as ICG (indocyanine green) are currently used in surgery [2,3], but these organic fluorophores are limited in terms of stability and are only used for direct imaging. In a recently developed imaging method, persistent luminescent particles are used, which are charged optically outside the body and are then injected [4]. While these particles keep emitting light for a long time in vivo without any additional excitation, such particles can emit only a limited amount of light, determined by the number of electronic traps that can be filled upon excitation [5]. To solve this limitation, it should be possible to excite the particles in vivo in the near-infrared exploiting the so-called biological windows (BWs) and using time-gated detection. In this way, the luminescent particles do not have to store the energy from the excitation, but can emit light right after excitation. There are three wavelength regions where there is an increased transparency of biological tissues where the excitation light can penetrate the body more deeply to reach the luminescent particles. The first biological window spans the wavelength range from 700 nm to 950 nm; the second biological window is found from 1000 nm to 1350 nm; and the third biological window covers the region from 1550 nm to 1870 nm [6,7]. In an ideal situation, both the excitation and emission would lie in a biological window, thus allowing both in vivo excitation and detection of the emitted light from the particles. Tissues show a strong autofluorescence when excited by NIR light [8]. This intrinsic autofluorescence has an emission up to 1200 nm, but its decay time is very short [9]. Therefore, when a delay time of 1 μs is applied between the excitation and the collection of the NIR emission, autofluorescence detection is avoided. This makes it preferable to use long lifetime phosphors for this application. If the phosphor has a lifetime of the order of 100 μs, there is ample timing for detecting the particle luminescence after the autofluorescence has decayed, using a time-gated detection setup.

In previous work [6,9], Nd-doped nanoparticles with long fluorescence lifetime were already proposed as suitable candidates for time-gated NIR bio-imaging. Indeed, one of the main advantages of Nd-doped phosphors is the fact that both excitation and emission lie within the biological transparency windows. Moreover, with the commercialization of affordable InGaAs cameras, allowing imaging beyond the 1000 nm wavelength limit of Si-based cameras, the second biological window has become easily accessible. This paper investigates the synthesis and properties of a Nd-doped phosphor, GdVO${}_{4}$. Apart from its near-ideal excitation and emission characteristics, GdVO${}_{4}$:Nd can also be used as an MRI probe due to its Gd magnetic host ions [10,11], thus allowing multimodal imaging with a single set of particles.

## 2. Materials and Methods

### 2.1. Synthesis

Phosphors were synthesized using high temperature solid state reaction in air in alumina crucibles. Two different solid state reactions were used.

- The first series was synthesized by using NH${}_{4}$VO${}_{3}$ (Alfa Aesar, 99.93%, CAS: 7803-55-6) and Gd${}_{2}$O${}_{3}$ (Alfa Aesar, 99.99%, CAS: 12064-62-9) as precursor materials. Here, the solid state reaction aimed for was:
  $$Gd_{2}O_{3}+2NH_{4}VO_{3}\to 2GdVO_{4}+H_{2}O+2NH_{3}$$
- The second series used a different precursor next to Gd${}_{2}$O${}_{3}$, namely V${}_{2}$O${}_{5}$ (Alfa Aesar, 99.99%, CAS: 1314-62-1). The solid state reaction aimed for was:
  $$Gd_{2}O_{3}+V_{2}O_{5}\to 2GdVO_{4}$$

The Nd dopant was added to the host precursors in the form of Nd${}_{2}$O${}_{3}$ (CAS: 1313-97-9). The precursors were weighed according to the correct stoichiometric ratio. For studying the Nd concentration dependence, two series of samples were synthesized. For the series using the NH${}_{4}$VO${}_{3}$ precursor (named GA samples), 0%, 0.5%, 1%, 2%, 5%, and 10% Nd concentrations were prepared, and for the series with the V${}_{2}$O${}_{5}$ precursor (named GV samples), 0%, 1%, 2%, 3%, 5%, and 7% Nd concentrations were selected. These were all atomic concentrations, aiming at specific mole fractions Nd/Gd. The precursors for the two series were heated to 800 °C for 1 h in an air atmosphere in a muffle furnace (Nabertherm LT 5/13) with a heating rate of 300 °C/h. Then, they were cooled to room temperature by natural cooling. Before heating again, the samples were ground in a mortar until a powder was obtained. They were reheated to 1100 °C for 3 h in air, with a heating rate of 300 °C/h to complete the reaction. After cooling down to room temperature by natural cooling, the samples were ground again prior to analysis.

To remove the V${}_{2}$O${}_{5}$ residue, two representative samples (GA1000 and GV1000, see below) were washed. These samples are designated with “_W”. Samples were washed by centrifugation– resuspension cycles (3500 rpm, 5 min) using NaOH 2 M aqueous solution (twice) and an acetone-water 1:1 mixture (twice). The powder was finally dried in an oven at 50 °C overnight.

### 2.2. Characterization Methods

#### 2.2.1. PXRD

PCRX (powder X-ray diffraction) was performed on a θ-2θ diffractometer (Siemens D5000) with Cu Kα radiation (λ = 0.15406 nm), with generator settings 40 kV and 40 mA. The data were recorded in the range from 2θ 15° to 90°, a 0.02° step size, and 3 s per step. From the position of the 13 largest peaks across the angle range and their corresponding Miller indices, the lattice constants of the crystallites were determined by least squares minimization.

#### 2.2.2. SEM and EDX

SEM (scanning electron microscopy) was performed to study the particle morphology, using a Hitachi S3400-N SEM, operating at low pressure (20 Pa), and an FEI Quanta 200 FEG SEM operating at high vacuum. For the FEI instrument, a thin gold layer was sputtered on the samples to counter the charging effect. Together with the Hitachi SEM, EDX (electron dispersive X-ray spectroscopy) was performed, using a Thermo Noran 7 detector, to allow identification of the elements present in the material. Since no suitable standards for these powder samples were available, only the ZAF (standard atomic number-absorption-fluorescence) corrections were applied for the calculation of the element concentrations.

#### 2.2.3. Decay Measurements

Luminescence decay measurements were performed by using an NT342 series tunable Nd:YAG pumped OPO laser system from Ekspla tuned to an excitation wavelength of 808 nm at 10 Hz. The luminescence intensity was measured using an amplified InGaAs photodiode detector with a bandwidth of 5 MHz (PDA20C from Thorlabs) coupled to a Rigol DS2302A 2 channel 300 MHz oscilloscope.

#### 2.2.4. Absorbance and Emission

Diffuse reflectance spectra were obtained using a Perkin Elmer Lambda 1050 UV-Vis-NIR spectrophotometer using an integrating sphere, with a wavelength resolution of 0.25 nm. The measured reflectance spectra were first normalized to 100% and then converted to absorbance spectra using the Kubelka–Munk transform [12].

The emission spectra where recorded using an Avantes fiber-coupled cooled InGaAs array spectrometer, Model AVASPEC-NIRS512-1.7-HSC-EVO. The excitation was performed using a continuous 808 nm/500 mW diode laser (Roithner Lasertechnik) in this case.

## 3. Results

After synthesis, the products did not have an entirely homogeneous body color. For the NH${}_{4}$VO${}_{3}$ precursor, the sample had a green/reddish color, and for the V${}_{2}$O${}_{5}$ precursor, the sample had a green/yellowish color. From experiments (diffuse reflectance spectra, shown in Section 3.2) it was found that some of the precursors did not properly react. For the NH${}_{4}$VO${}_{3}$ sample, V${}_{2}$O${}_{5}$ was formed due to the high temperature according to the following reaction [13]:

$$2NH_{4}VO_{3}\to V_{2}O_{5}+2NH_{4}H_{2}O$$

To investigate the washing dependence of the samples, two samples were made at 1000 °C for 2 h in an air atmosphere in a muffle furnace (Nabertherm LT 5/13), using a heating rate of 300 °C/h.

### 3.1. Structural Properties

#### 3.1.1. PXRD

**Figure 1:**
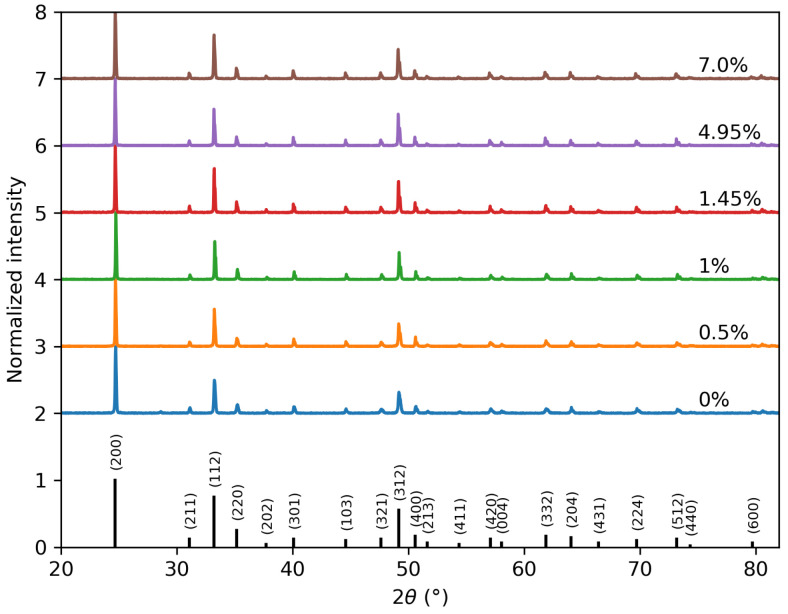
PXRD pattern of GdVO${}_{4}$ from all the concentrations of the GA series. Concentrations are those obtained by the EDX measurements except for 0%, 0.5%, and 1%. Bottom: expected lines (GdVO${}_{4}$, PDF number: 00-017-0260).

**Figure 2:**
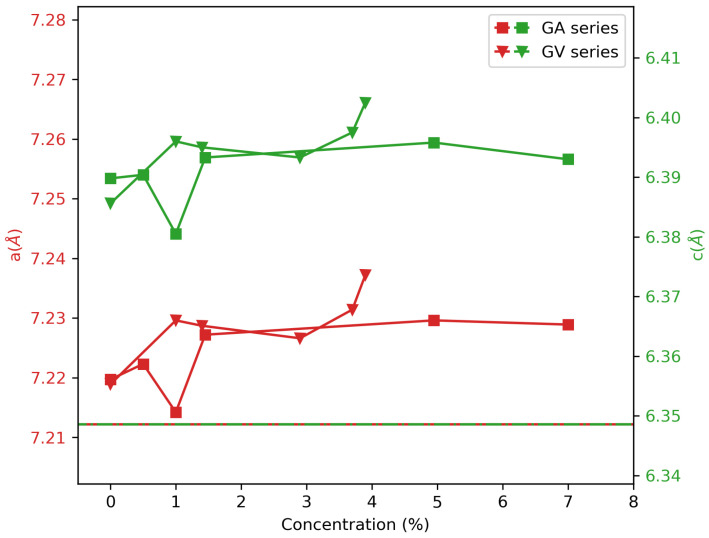
Lattice constants as obtained from the fit of the position of the XRD peaks. Concentrations are those obtained by the EDX measurements except for 0%, 0.5%, and 1%. The horizontal line shows the database values of the lattice constants.

Typical XRD patterns are shown in Figure 1 for the GA series, synthesized with the ammonium vanadate precursor. Those XRD patterns show the same peaks and shapes and indicate the presence of a single tetragonal zircon-type phase of GdVO${}_{4}$ matching with the PDF (powder diffraction file) number 00-017-0260. In the XRD pattern, no signal is found from V${}_{2}$O${}_{5}$, which means that if there is V${}_{2}$O${}_{5}$ present in the sample, it has to be amorphous. The minor peak at around 28°, visible in the undoped sample, could be traced back to a small fraction of unreacted Gd${}_{2}$O${}_{3}$. In Figure 2, the lattice parameters are found from a least squares fit of the position of 13 different peaks across the angle range. Nd doping only slightly influenced the XRD patterns. Nd has a slightly larger ionic radius than Gd (1.109 Å and 1.053 Å, respectively, for eight-fold coordination [14]). Therefore, it is expected that at higher concentrations of Nd, the lattice slightly expands, and the diffraction peaks shift towards lower angles. As seen in Figure 2, this effect is very limited and is dominated by an overall deviation of the lattice constants compared to the reported values from the PDF file. Remarkably, the *c* parameter shows a larger deviation than the *a* parameter, even for the undoped sample. To determine the origin of this offset, further experiments are needed; apparently, there is a combined effect from the synthesis procedure and from Nd doping. There are no structural differences between the samples that were made using the two precursors with the same dopant concentration.

#### 3.1.2. SEM

**Figure 3:**
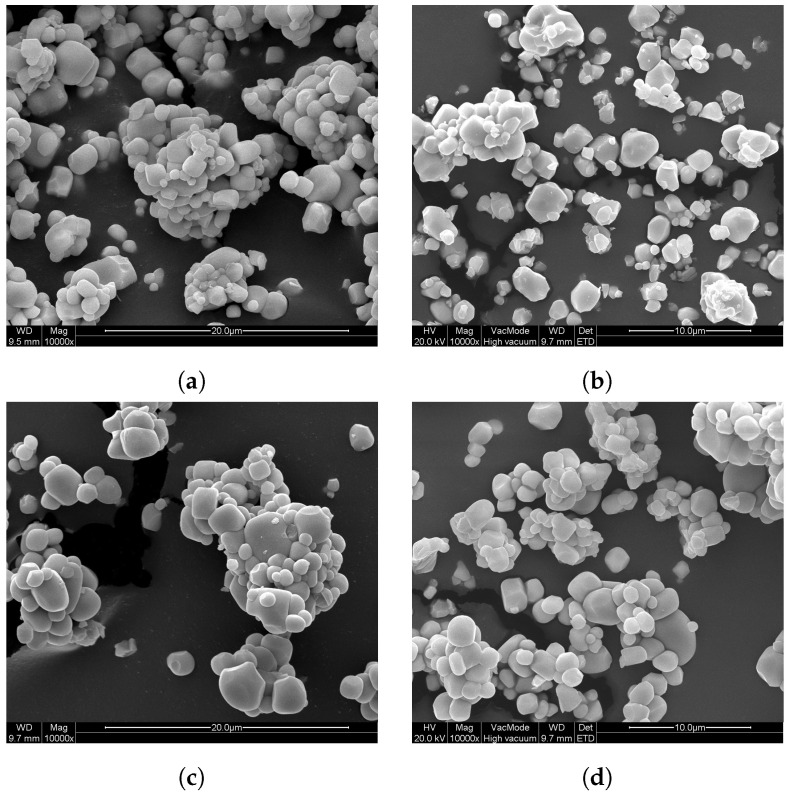
SEM images: (**a**) GA1000 sample, (**b**) GA1000_W sample, (**c**) GV1000 sample, and (**d**) GV1000_W sample. All scales are equal.

SEM images (Figure 3) show polyhedral particles with a size of around 1 μm, which form agglomerates. The EDX spectra confirmed the incorporation of the intended amount of Nd in the phosphor (measured on the GA1000 sample). From the SEM images in Figure 3, there seems no difference in particle size or particle shape between the GA1000 and GV1000 sample. The particle shape does not change upon washing, but the particles become less agglomerated.

**Figure 4:**
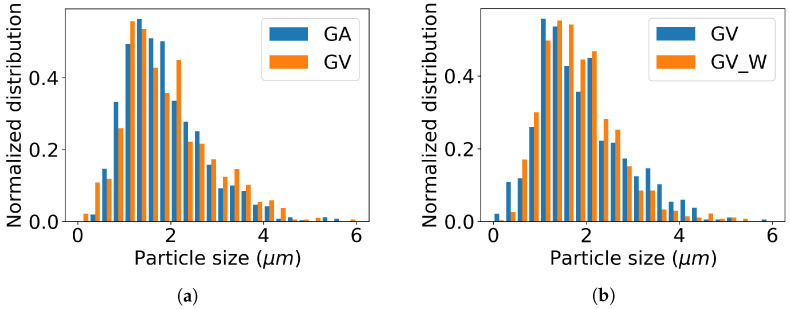
Particle size distribution of (**a**) the GA1000 and GV1000 samples and (**b**) the GV1000 and GV1000_W samples.

In Figure 4, the distribution of the particle size is shown as measured from SEM images of a large distribution of particles of the GA1000 samples. The particles were measured by taking the maximum diameter. All the particles have sizes in the range 0.2–6 μm. The same distribution is found for the GV1000 samples. The mean particle size of the measured samples can be found in Table 1.

#### 3.1.3. EDX: Element Distribution

Energy dispersive X-ray analysis was performed on the two series of samples. In every sample, it was clear that it mostly consists of Gd, V, and O. In Table 2, the measured concentrations of Nd, Gd, V, and O for the GA series and GV series are found. The Nd/Gd concentration was calculated from the L lines of Nd and Gd. The L lines for Gd are at: 8.3756 keV, 7.9303 keV, and 7.24218 keV [15] and for Nd: 7.1260 keV, 6.7215 keV, and 6.2079 keV [15]. This edge is chosen because they are the strongest ones that could be measured. In Table 2, the concentrations are the averages of two measurement, and N.D. means that the concentration fell below the detection limits. This is due to the fact that the lines of Gd and of Nd lie close to each other and partly overlap. The washing procedure did not decrease the Nd concentration and did not change the ratio Gd/V. Furthermore, the Nd content is consistent with the nominal amount for all samples up to 5%, then a significantly lower content is observed. It is clear that at higher Nd precursor concentrations, not all Nd is properly incorporated in the host lattice. Nevertheless, some additional Nd is still built in when trying to incorporate more than 5%, as seen from both the EDX results and the shortening of the decay times (see below).

### 3.2. Optical Properties

**Figure 5:**
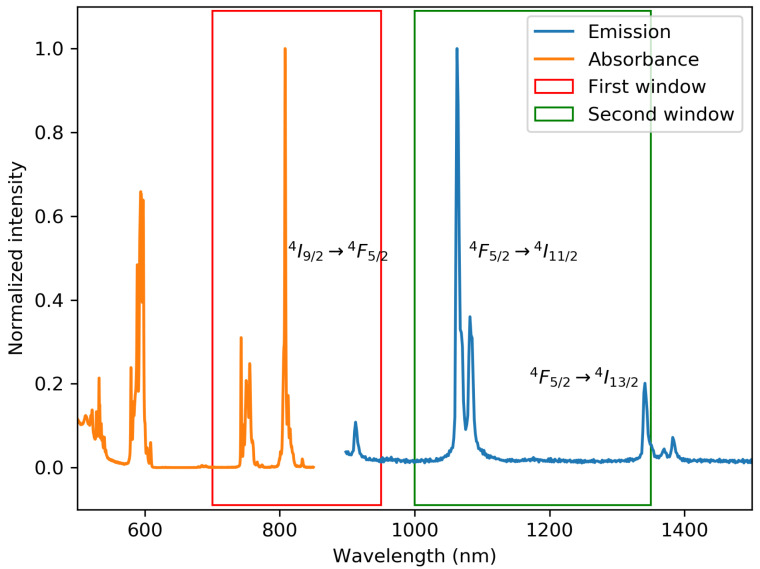
Absorbance and emission of the GA2 sample. The red rectangle shows the first optical window, and the green rectangle shows the second optical window of the biological tissue. The excitation source was a 500 mW 808 nm diode laser.

In Figure 5, the absorbance and emission of a typical sample are given, together with the first and second optical transparency windows for biological tissue. As for most phosphors with Nd, there is a strong absorption at around 808 nm (${}^{4}I_{9/2}$→${}^{4}F_{5/2}$), in the first optical transparency window for biological tissue. The emission has a strong peak between 1045 nm and 1080 nm (${}^{4}F_{3/2}$→${}^{4}I_{11/2}$) and a smaller one just below 1350 nm (${}^{4}F_{3/2}$→${}^{4}I_{13/2}$) [11,16]. These lines are split due to the Stark effect [17]. Furthermore, del Rosal et al. stated that GdVO${}_{4}$ has better performance with respect to other hosts, in terms of modulating the excitation wavelength (many other hosts give rise to an excitation wavelength close to 790 nm) [18].

#### 3.2.1. Washing Dependence

**Figure 6:**
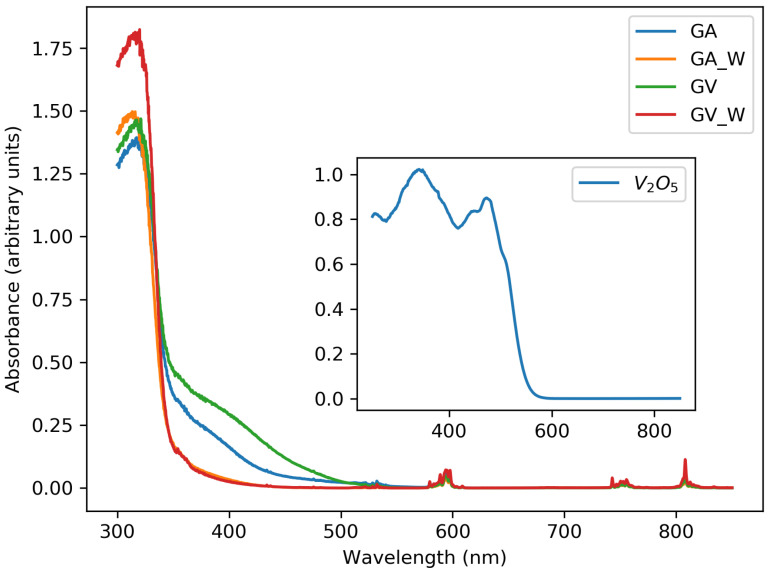
Influence of the washing procedure on the optical absorption of samples without and with a washing procedure. The GA1000 and GV1000 samples are shown. The inset shows the absorption of pure V${}_{2}$O${}_{5}$ powder for comparison.

The samples with the V${}_{2}$O${}_{5}$ precursor had a yellow/brownish body color, and the samples with the NH${}_{4}$VO${}_{3}$ precursor were reddish after synthesis (consistent with the absorbance spectra in Figure 6). From this body color of the samples, it was assumed that not all the precursors had reacted or that secondary phases were formed. To remove the remaining precursors from the samples, the samples were washed as described in Section 2.1. The absorption below 500 nm was at least partly attributed to band gap absorption of V${}_{2}$O${}_{5}$. V${}_{2}$O${}_{5}$ is a direct band gap semiconductor with a band gap of 2.41 eV [19], corresponding to an absorption edge at around 514 nm. Even a low amount of V${}_{2}$O${}_{5}$ can thus lead to the strong coloration observed. The only indication of the presence of V${}_{2}$O${}_{5}$ (and possibly other unreacted products) is the sample color and the corresponding absorption below 500 nm. We did not observe any trace of V${}_{2}$O${}_{5}$ in XRD or SEM/EDX. We therefore assume it is only present as a trace impurity.

Looking at Figure 6, there is clearly a difference in absorption between the washed samples and unwashed samples. The spurious absorption from 350 nm to 500 nm disappeared, and the two samples with different precursor yield a similar result after washing. Before the washing procedure, there is also more absorption between 350 and 500 nm when the starting precursor is V${}_{2}$O${}_{5}$. The strong absorption band that starts at 350 nm is the charge transfer (CT) band O${}^{2-}$–V${}^{5+}$ of the GdVO${}_{4}$ host [20].

**Figure 7:**
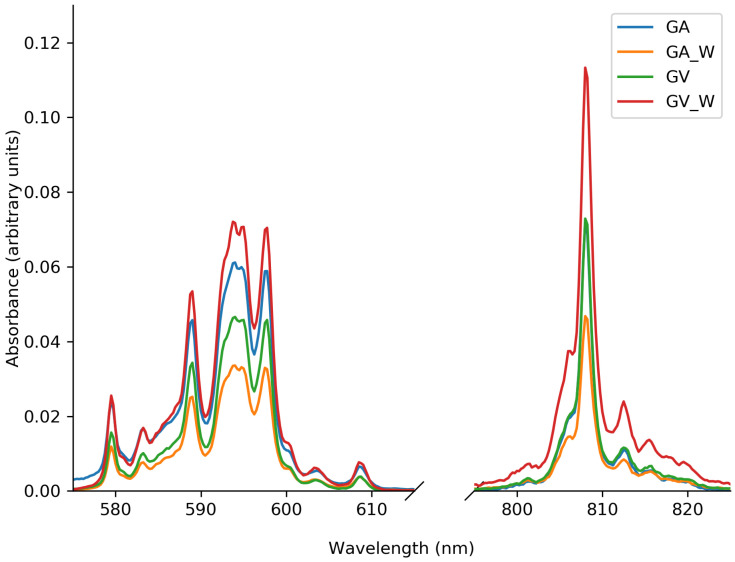
Detail of the Nd-related absorption features of Figure 6.

The Nd-related absorption peaks (Figure 7) have the same shape, but there is a slight difference between samples before and after the washing procedure. For the GA1000 sample, the absorbance decreases; meanwhile, with the GV1000 sample, the absorbance is higher after washing. The diffuse reflectance measurements are consistent with the white body color of the samples after washing. Deriving absolute absorbance values from diffuse reflectance measurements using an integrating sphere is notoriously difficult, and probably prone to some error. There is no clear physical reason why the Nd-related absorption would change upon washing; as described below, this effect is largely dominated by the effect of doping concentration.

**Figure 8:**
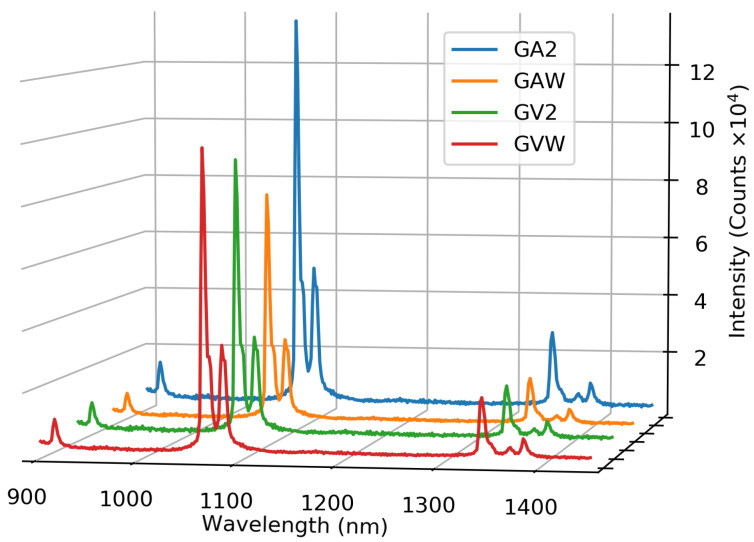
Emission spectrum of the unwashed and washed samples. Excitation with a 500 mW 808 nm laser.

**Figure 9:**
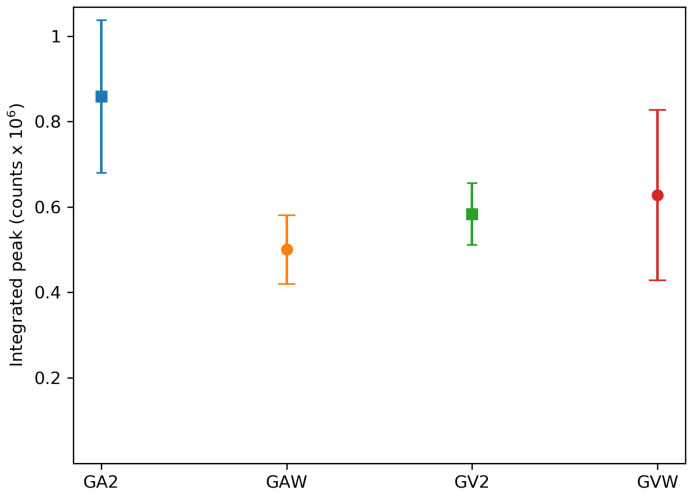
Integrated peak intensity from 1050 nm to 1100 nm corrected for the background for the unwashed and washed samples.

The emission of the samples can be seen in Figure 8, upon excitation with a 500 mW 808 nm diode laser. All samples have the same emission peaks, but slightly different peak intensities. The relative emission intensity of the samples was calculated by integrating the emission spectra in the range 1050 nm to 1100 nm and subtracting the background, as calculated from the integrated intensity in the range 1200 nm to 1250 nm, where no emission is observed. These intensities are the average of three measurements and can be seen in Figure 9. The intensity of the washed samples is higher for the GV1000 sample and is lower for the GA1000 sample. For the GV sample, this difference is quite small, but these intensities are consistent with the difference in absorbance. For the GA sample, the washed one has a lower absorption and so a lower emission, but for the GV sample, the washed one has a higher absorption and so a higher emission. Overall, the effect of washing on the emission intensity of the samples is limited; therefore, washing is not essential in order to obtain strongly emitting samples.

**Figure 10:**
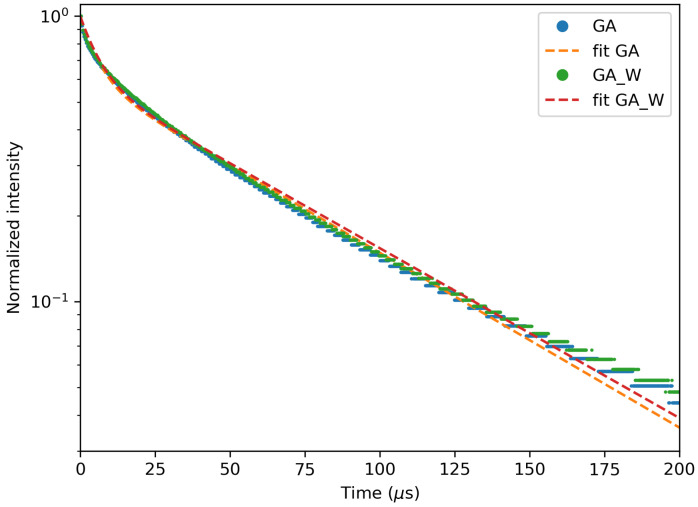
Decay measurement of GA1000 with 2% Nd before and after washing, with their fit.

In Figure 10, the decay measurement for the unwashed and washed GA1000 sample is shown with the corresponding fit. A double exponential is fitted to the data, and a fast and slow component can be extracted. The fitting results are summarized in Table 3 with the total contribution of the short decay component to the integrated intensity. The decay times of the washed samples are slightly longer than the decay times of the unwashed samples. For the GA sample, the decay time increased from 70.9 μs to 73.0 μs, and for the GV sample, we could see an improvement from 72.6 μs to 81.9 μs.

#### 3.2.2. Nd Concentration Dependence

For the study of the concentration dependence, two series of samples were made, namely the GA series with Nd concentrations of 0.5%, 1%, 2%, 5%, and 10% and the GV series with Nd concentrations of 1%, 2%, 3%, 5%, and 7%. Like the washing dependence, diffuse reflectance, emission, and decay are studied.

**Figure 11:**
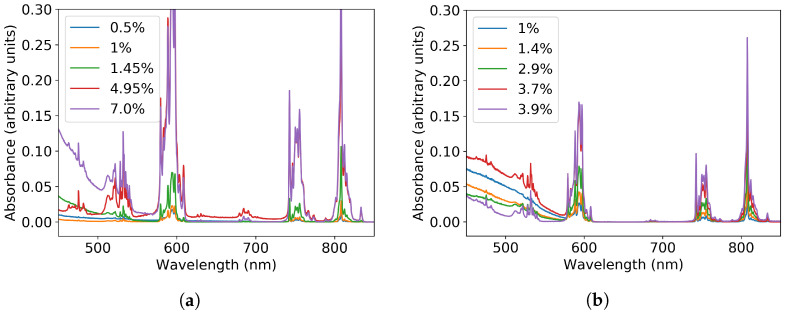
Absorbance for the (**a**) GA series and (**b**) GV series where the concentrations are those from the EDX measurements except for 0.5% and both 1% samples.

In Figure 11, we can find the absorbance spectra as a function of wavelength for different concentrations of Nd for each series. At high concentrations, the maximum absorbance of the Nd-related peaks is almost independent of the concentration. It seems that at 5%, the maximum absorbance is achieved for the Nd-related peaks. Between 350 and 500 nm, the absorbance by V${}_{2}$O${}_{5}$ is again observed, which can be solved by washing the samples as shown previously. The broad band absorption in the near-UV spectral region is again assigned to the CT band of GdVO${}_{4}$, as seen in the washed samples [20].

**Figure 12:**
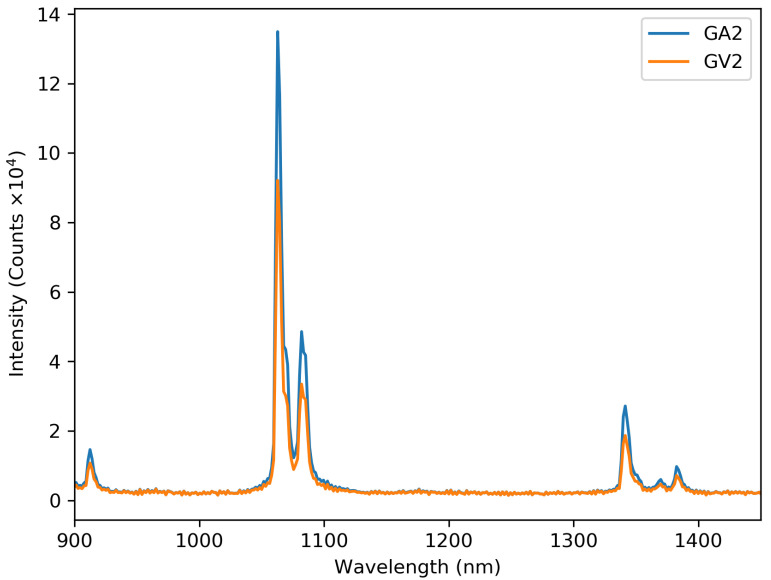
The recorded emission spectra of GA2 and GV2. Excitation with a 500 mW 808 nm laser.

**Figure 13:**
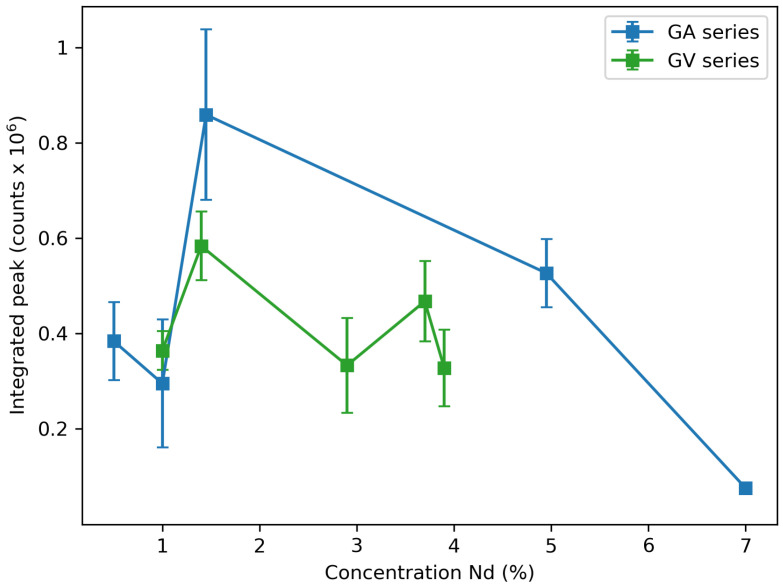
Integrated peak intensity from 1050 nm to 1100 nm corrected for the background for the GA series and the GV series. Concentrations are those obtained by EDX measurements except for the 0.5% and both 1% samples. Measured intensities are the average of three measurements on different samples, and the error bars reflect the resulting standard deviation.

The emission spectra of GA2 and GV2 can be seen in Figure 12, where the excitation was performed using a 500 mW 808 nm laser. The positions of the peaks are the same for all other samples, while only intensity changes were observed. To investigate the concentration dependence of the emission, Figure 13 shows the integrated intensity of the highest peak (1050 nm to 1100 nm) corrected for the background. The 2% sample has the highest emission intensity recorded. At higher concentrations, the emission drops due to concentration quenching.

**Figure 14:**
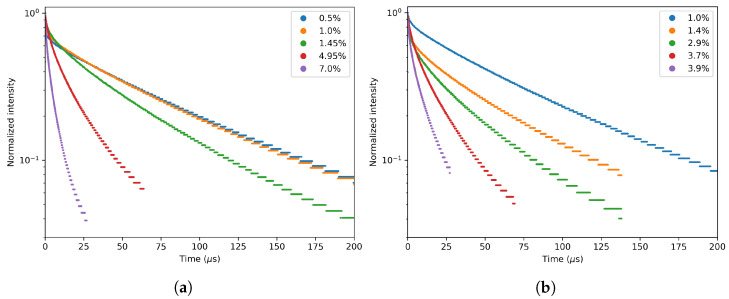
Decay measurement of (**a**) the GA series and (**b**) the GV series. The decay times fitted through these graphs can be found in Table 3. The concentrations are those found in the EDX measurements except for the 0.5% and 1% samples.

**Figure 15:**
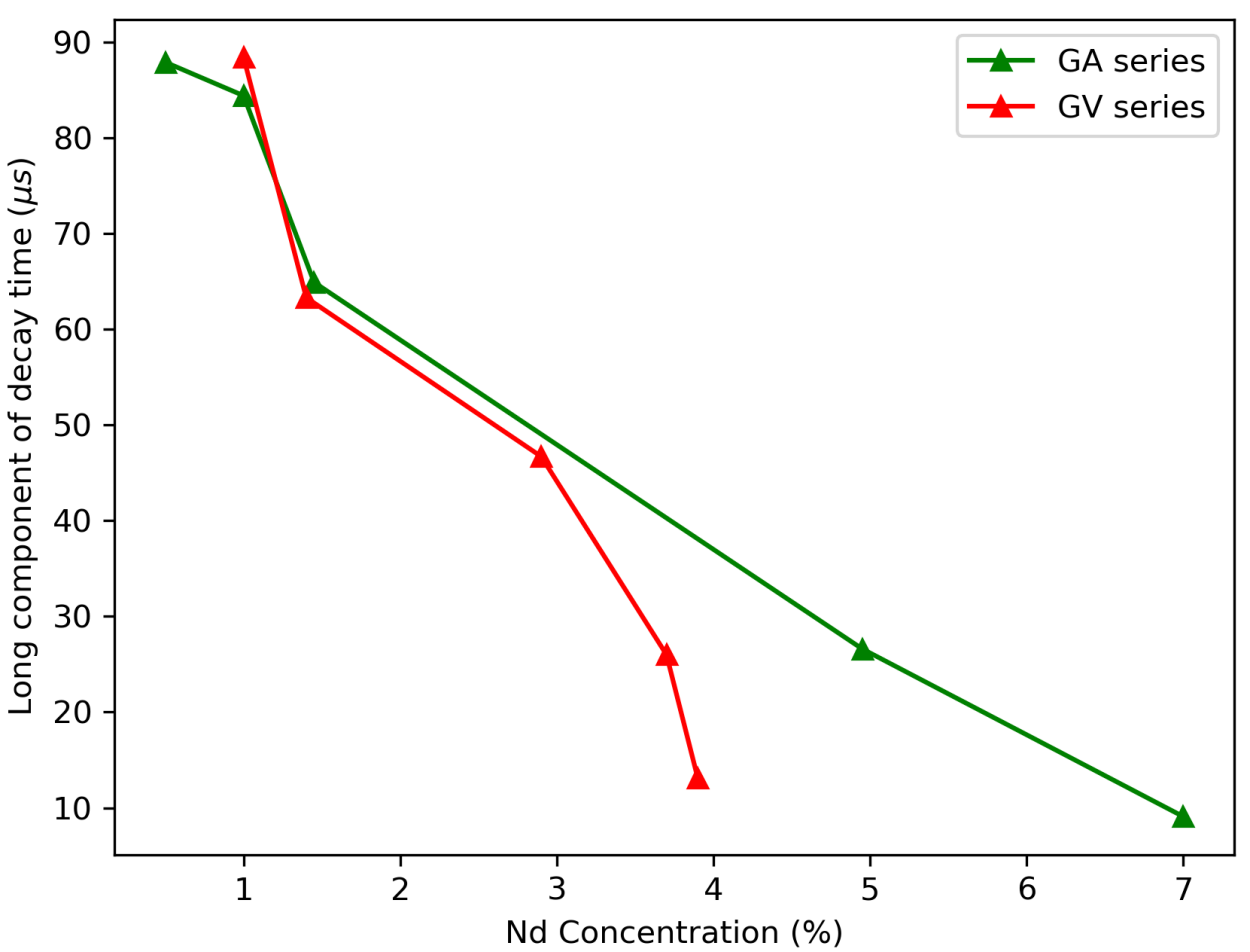
Long decay components as a function of the concentration for the two series (GV and GA) as a function of Nd concentration. The concentrations are those found in the EDX measurements except for the 0.5% and both 1% samples.

In order to use the GdVO${}_{4}$ as an in vivo imaging tracer for time-gated imaging, the decay time must be long enough to overcome the autofluorescence. The decay times obtained in this work can be found in Table 3 and the corresponding decay curves in Figure 14 and Figure 15. From these figures, it is clear that the longest decay times are found for the lowest dopant concentrations. This effect is also related to concentration quenching, where the excited energy has a higher probability to decay non-radiatively, which lowers the decay times. The differences between precursors are negligible when we are comparing two samples with the same dopant concentration. For in vivo time-gated imaging, every concentration lower than 5% can be used if we take only the decay time into account. The first μs is lost due to time-gated imaging when we wait until the autofluorescence has decayed [9]. The decay times obtained in this work are consistent with decay times found in the literature. Jensen et al. found 90 μs for a 1.2% Nd dopant concentration [21], and Ogawa et al. found 44 μs for a 2% Nd dopant concentration [22].

## 4. Discussion

### 4.1. Washing Dependence

Washing the sample can remove all the amorphous V${}_{2}$O${}_{5}$ present as a reaction residue. The absorption from 350 to 500 nm hereby disappeared, but the absorption peak at 808 nm, which is essential for the bio-imaging application, did not change appreciably in intensity. For the GV1000 sample, the absorption peak increased; meanwhile, for the GA1000 sample, it decreased. The same behavior was observed for the emission. The GV1000 sample showed an increased intensity, but the GA1000 sample decreased in emission intensity. The decay time showed a small increase upon washing. These effects are all quite small; the dopant concentration has a much larger influence on the optical properties. Therefore, washing can be performed, but the effect on the most important optical properties for the in vivo imaging is small. Therefore, the washing procedure can be omitted, unless the remaining reaction products and amorphous V${}_{2}$O${}_{5}$ fraction would be detrimental for the physical and toxicological characteristics of the material at a further stage of the research. V${}_{2}$O${}_{5}$ can cause health issues by damaging DNA inside cells [23], which might be a good reason to perform a washing procedure for any bio-imaging application.

### 4.2. Concentration Dependence

The samples with a 5% dopant concentration showed the highest absorbance, but concentration quenching was already observed; therefore, lower concentrations are optimum. From the decay profiles, the 0.5% and 1% samples showed the best results because they had the longest decay time and lowest loss in intensity the first μs when time-gated imaging was applied. Taking the absorption, the integrated peak emission, and decay time into account, the 2% samples are best suited, because they have the highest emission intensity combined with a decay time that is long enough to counter the autofluorescence that is induced by the excitation pulse.

## 5. Conclusions

The solid state synthesis of Nd-doped GdVO${}_{4}$ was performed using two different precursors, both leading to highly efficient phosphors. Because not all the precursors reacted in the solid state reaction and there was the formation of amorphous V${}_{2}$O${}_{5}$ with the NH${}_{4}$VO${}_{3}$ precursor due to the high temperature, additional absorption below 500 nm was observed, but this did not substantially decrease the phosphor performance. The V${}_{2}$O${}_{5}$ could be removed by washing the precursor away from the as-prepared phosphor. The washed samples did not perform any better or worse compared to the unwashed samples; however, the toxic precursors were removed, and the material was more homogeneous and showed a white body color after washing.

A dopant concentration between 1% and 2% was found to be optimum for the intended application. From the diffuse reflectance spectra, it was seen that the absorption was optimum at a 5% Nd concentration, but the decay time was lower for these high concentrations, due to concentration quenching. To obtain a long lifetime phosphor, the 1% doped samples, with a decay time of around 85 μs, or the 2% samples, with a decay of around 60 μs, would be best. The emission intensity was similar for these samples.

There were no significant differences between samples prepared using the different precursors. Since V${}_{2}$O${}_{5}$ leads to a lower amount of V${}_{2}$O${}_{5}$ in the as-prepared product after synthesis, the latter precursor was preferred.

The cytotoxicity and biodistribution of particles, to be used in bio-imaging, are highly dependent on particle size and particle surface functionalization. In the present work, the particles are still micron-sized, and too large for bio-imaging as such. Both the preparation of smaller particles, their surface functionalization, and testing for biocompatibility are the subject of our follow-up research.

## Figures and Tables

**Table 1:** Mean particle size (μm) of the GA1000, GA1000_W, GV1000, and GV1000_W samples, obtained by measuring on a large distribution of particles (more than 1000) on the SEM images.

Mean Particle Size (μm)	Unwashed	Washed
GA samples	1.88	1.35
GV samples	1.91	1.83

**Table 2:** EDX concentrations (at%) in the samples from the GA and GV series. N.D. means that the concentration fell below the detection limit.

	Synthesis Nd/Gd (%)	Nd/Gd (%)	Nd (%)	Gd (%)	V (%)	O (%)
GA series	0.5	N.D.	N.D.	23.5	25.2	51.3
	1	N.D.	N.D.	23.1	24.9	51.9
	2	1.45	0.3	20.8	22.6	56.3
	2 (W)	1.8	0.3	17.7	19.2	62.7
	5	4.95	1.1	22.2	24.8	51.8
	10	7.4	1.4	19.4	22.8	56.3
GA series	1	N.D.	N.D.	22.0	23.8	54.2
	2	1.4	0.3	21.9	23.7	54.1
	2 (W)	1.6	0.3	19.3	21.0	59.4
	3	2.9	0.6	21.6	24.1	53.6
	5	3.7	0.8	20.7	23.4	55.1
	7	3.9	0.8	20.3	23.1	55.8

**Table 3:** Decay times of all the samples.

Sample	$\tau_{1}\left(\mu s\right)$	$\tau_{2}\left(\mu s\right)$	Fraction Short Component (%)
GA1000_2	70.9	5.8	5.03
GA1000_2 _W	73.0	6.5	5.49
GV1000_2	72.6	4.7	4.39
GV1000_2 _W	81.9	6.3	5.81
GA series			
GA05	87.9	0.7	0.46
GA1	84.4	1.4	0.90
GA2	64.9	3.8	3.37
GA5	26.6	3.5	9.43
GA10	9.08	1.2	13.7
GV series			
GV1	88.4	5.8	2.21
GV2	63.3	1.5	1.63
GV3	46.7	2.0	3.45
GV5	26.0	3.1	8.54
GV7	13.1	1.2	6.69
